# High curative resection rate with weekly cisplatin, 5-fluorouracil, epidoxorubicin, 6S-leucovorin, glutathione, and filgastrim in patients with locally advanced, unresectable gastric cancer: a report from the Italian Group for the Study of Digestive Tract Cancer (GISCAD)

**DOI:** 10.1038/sj.bjc.6601752

**Published:** 2004-03-30

**Authors:** S Cascinu, M Scartozzi, R Labianca, V Catalano, R R Silva, S Barni, A Zaniboni, A D'Angelo, S Salvagni, G Martignoni, G D Beretta, F Graziano, R Berardi, V Franciosi

**Affiliations:** 1 Clinica di Oncologia, Università Politecnica delle Marche-Azienda Ospedaliera Umberto I, via Conca, Ancona 60020, Italy; 2 Medical Oncology, Ospedali Riuniti, Bergamo, Italy; 3 Medical Oncology, Azienda Ospedaliera S Salvatore, Pesaro, Italy; 4 Medical Oncology, Ospedale di Fabriano, Italy; 5 Medical Oncology, Azienda Ospedaliera di Treviglio, Italy; 6 Medical Oncology, Casa di Cura Poliambulanza, Brescia, Italy; 7 Medical Oncology, Azienda Ospedaliera-Universitaria, Parma, Italy; 8 Medical Oncology, Azienda Ospedaliera S Carlo Borromeo, Milano, Italy; 9 Medical Oncology, Ospedale di Urbino, Italy

**Keywords:** gastric cancer, preoperative chemotherapy, curative resection, neoadjuvant

## Abstract

The aim of the present study was to evaluate the role of a weekly preoperative chemotherapy in locally advanced, unresectable gastric cancer. In all, 82 patients with an Eastern Oncology Cooperative Group PS ⩽2 and normal cardiac function were enrolled onto the study. Surgical unresectability was confirmed in 52 patients (63%) at laparotomy, and in 30 (27%) cases by CT scan of the abdomen and endoscopic ultrasonography. Chemotherapy treatment was: cisplatin 40 mg m^−2^; 5-fluorouracil 500 mg m^−2^; epidoxorubicin 35 mg m^−2^; 6S-leucovorin 250 mg m^−2^ and glutathione 1.5 g m^−2^ (PELF). One cycle consisted of 8 weekly treatments. Response to chemotherapy was observed in 40 of 82 patients (49%): six (7%) complete and 34 (41%) partial responses, and in four (5%) cases a complete pathological response was confirmed. Of the 40 responding patients, 37 (45%) had potentially curative surgery. Grade 3/4 leucopenia and thrombocytopenia occurred in three and two patients. At a median follow-up of 48 months, 25 of the 37 resected patients (68%) were alive and 24 (65%) were disease free. The median and 4-year survival for the whole group was 17 months and 31%, respectively. The median survival was 12 months for inoperable patients and it was not reached in resected patients.

Although the incidence of gastric cancer has gradually decreased in many Western Countries, it remains one of the leading causes of cancer-related deaths worldwide, and it now ranks second only to lung cancer with about 755 000 new cases per year (Karpeh *et al*, 2001). Since screening for early detection is not performed in Western Countries, in approximately 50% of newly diagnosed cases, the tumour is beyond its local–regional margins (Kelsen, 1996; Karpeh *et al*, 2001). Surgery remains the mainstay of any curative treatment, but only when a radical resection is feasible (removal of all gross cancer cells at the resection margins as determined by histopathological examination). Those patients who are considered not amenable of curative resection generally receive chemotherapy in order to obtain palliation of symptoms and improved survival. Since there is no evidence that a more aggressive treatment could result in a better survival, most of the patients receive a combination of 5-fluorouracil (5-FU), mitomycin C or cisplatin (Karpeh *et al*, 2001). Only a few studies have focused on the role of preoperative chemotherapy in unresectable gastric cancer. Comprehensively, these trials suggested that chemotherapy could allow radical surgery in approximately 40% of all cases not amenable of curative resection at presentation, but at the cost of severe toxicity (Kelsen, 1996). In a pilot trial, we observed that a preoperative chemotherapy with weekly cisplatin (CDDP), epidoxorubicin (epi-ADR), 5-FU, 6S-leucovorin, glutathione and bone marrow support (filgastrim) could allow a radical resection in 13 out of 32 (41%) patients previously considered unresectable (Cascinu *et al*, 1998). These encouraging findings followed our demonstration of activity (62% overall response rate in 105 patients) of this chemotherapy regimen in patients with advanced gastric cancer. In this latter trial, five of 11 (45%) patients with exclusively locally advanced unresectable disease could undergo a curative resection after chemotherapy (Cascinu *et al*, 1997). In order to test whether the hypothesis of a more aggressive and expensive approach in this subset of gastric cancer patients could be justifiable, we prospectively analysed the effects of this intensive weekly treatment in a larger group of gastric cancer patients not amenable of curative resection.

## MATERIALS AND METHODS

### Patients’ selection

Patients with previously untreated, histologically confirmed, locally confined, gastric adenocarcinoma were eligible for the study, only after the primary tumour was considered not amenable of surgical resection. Unresectable disease was defined jointly by a medical oncologist, a gastroenterologist and an abdominal surgeon on the basis of the laparotomy findings and/or CT scan images (tumour size >7 cm, invasion of adjacent structures, such as pancreas, omentum, aorta, oesophagus, liver), endoscopy and endoscopic ultrasonography (EUS). Patients with distant, liver and peritoneal metastases were excluded. The American Joint Committee on Cancer staging system (6th edition) was applied. Patients were also required to have an Eastern Oncology Cooperative Group (ECOG) performance status ⩽2, an adequate hepatic (serum bilirubin <1.5 mg dl^−1^), renal (serum creatinine <1.5 mg dl^−1^) and bone marrow function (white blood cell (WBC) >4000 cells *μ*l^−1^ with an absolute granulocyte count >1500 cells *μ*l^−1^, platelet count >100 000 cells *μ*l^−1^) and aged between 18 and 70 years. As a potential cardiotoxic drug (epi-ADR) was included in the chemotherapy regimen, patients with a New York Heart Association class >2 were excluded from the study. Pretreatment assessment included complete blood cell count with WBC differential and platelet count, biochemical screening profile, serum creatinine and/or creatinine clearance, CT scan or radiograph of the chest, CT scan of the abdomen, endoscopy and a bone scan. Gated pool scan was not routinely performed unless the patient had a history of cardiac disease, in which case it was mandatory and the patient was ineligible for the study if the left ventricular ejection fraction was <45%. The protocol was approved by the institutional review board, and all patients gave informed consent, which indicated that they were fully aware of the investigational nature of the study itself.

### Chemotherapy

**Figure 1:**
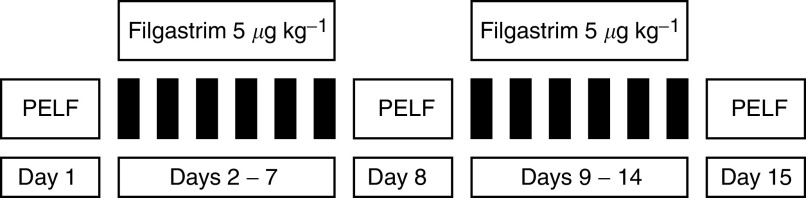
Diagram showing the treatment given in the first 2 weeks of therapy. The same treatment schedule was administered for 8 consecutive weeks.

All patients underwent chemotherapy according to the weekly PELF regimen, which consisted of a once a week administration of CDDP 40 mg m^−2^ as a 30 min infusion in 250 ml of normal saline, 5-FU 500 mg m^−2^ as a 15 min infusion in 100 ml of normal saline and epi-ADR 35 mg m^−2^ by intravenous bolus injection. A dose of 250 mg m^−2^ of 6S-stereoisomer of leucovorin as a 4 h infusion in 250 ml of normal saline was administered concurrently with hydration and glutathione 1.5 g m^−2^ in 100 ml of normal saline over 15 min, which was infused before each cisplatin administration in order to prevent CDDP-related neurotoxicity (Figure 1

). Antiemetic treatment with dexamethasone 20 mg and ondansetron 8 mg both given intravenously was administered, respectively, 45 and 15 min before CDDP infusion. At 2 h before CDDP administration, patients received intravenous hydration with 1500 ml of normal saline plus 20 mEq of potassium chloride and 15 mEq of magnesium sulphate. Intravenous fluids administration proceeded for 2 h after CDDP with 1000 ml of normal saline. All patients received filgastrim 5 *μ*g kg^−1^ by subcutaneous injection from the day after to the day before each chemotherapy administration. One cycle of chemotherapy consisted of 8 weekly chemotherapy administrations. Patients only received 8 weeks of treatment (one cycle). In the event of toxicity, chemotherapy administration was delayed by a week or until full recover in case of WBC count <4000 cells *μ*l^−1^, platelet count <100 000 cells *μ*l^−1^ or if grade 2 and 3 mucositis and diarrhoea occurred. The criteria for patient withdrawal from study were patient's refusal, tumour progression and any grade 4 toxicities. Chemotherapy was administered as an outpatient procedure in all cases. The planned delay between the last week of treatment and surgery was 6–8 weeks.

### Evaluation of response and toxicity

Objective response to chemotherapy was assessed after 8 weeks of therapy, combining findings from both CT scan of the abdomen and endoscopy, including a new biopsy of the tumour, if still visible, or a biopsy of the area originally involved by the tumour. Endoscopy and endoscopic ultrasonography was performed when clinically indicated. Partial response (PR) was defined as having both CT scan evidence of PR, according to the World Health Organisation (WHO) criteria, and endoscopy showing a >50% reduction of the visible tumour, or complete disappearance of the tumour, but positive histology on biopsy of the previously involved area. Complete response (CR) was defined as a complete disappearance of the tumour as seen by CT scan of the abdomen and a complete resolution of the endoscopic findings without histological evidence of neoplastic cells on biopsy of the original site of the tumour. Toxicity was evaluated weekly according to the National Cancer Institute Common Toxicity Criteria (NCICTC). The decision to perform a laparotomy with the aim of a radical excision was evaluated each time a complete removal of the tumour was jointly judged feasible by a medical oncologist, a gastroenterologist and an abdominal surgeon.

## RESULTS

In all, 82 patients were enrolled onto this study by coinvestigators from seven Italian centres. The median age at diagnosis was 57 years (range 29–68 years); 57 (69.5%) patients were male and 25 (30.5%) were female, most of the patients enrolled were in good general condition as PS (ECOG) was 0–1 in 72 (88%) patients and two in the remaining 10 (12%) patients. The primary tumour was located in the body of the stomach in 40 (49%) cases, in the gastro-oesophageal junction in 18 (22%) cases, in the distal stomach in 18 (22%) cases and in the proximal stomach in six (7%) cases. In all, 52 (63%) patients underwent laparotomy, as a part of a failed attempt at radical primary surgery, before study entry, in these cases laparotomy confirmed the presence of locally advanced, unresectable disease, whereas in the remaining 30 (37%) patients the diagnosis of locally advanced disease was confirmed by CT scan of the abdomen (23 patients) and EUS (seven patients). All patients received eight weekly treatments (one cycle of chemotherapy). Patient characteristics are summarised in Table 1

**Table 1:** Patient characteristics

No of patients	82
*Age (years)*	
Median	57
Range	29–68
	
*Sex*	
Male/female	57/25
	
*PS (ECOG)*	
0	39
1	33
2	10
	
*Sites of primary tumor*	
Gastro-oesophageal junction	18
Proximal stomach	6
Body	40
Distal stomach	18
	
*Initial stages*	
T4 N0 M0	13
T4 N1-2 M0	51
T3 N1-2 M0	18
	
*Laparotomy*	
Yes	52
No	30

COG=Eastern Oncology Cooperative Group.

.

We observed a response to chemotherapy in 40 of 82 patients (49%), six (7%) patients had a CR and 34 (41%) had a PR. Of these, 30 (36%) patients showed disease stabilisation, whereas 12 (15%) patients progressed on chemotherapy. Among the 40 responding patients, 37 (45%) had potentially curative radical surgery and three (4%) were found not resectable at laparotomy. In four (5%) cases, a complete pathological response was confirmed (Table 2

**Table 2:** Stage at surgery (37 patients)

**Stage**	**Patients**
pT0N0	4
pT1N0	1
PT2N0	2
PT3N0	12
PT2N1	5
PT3N1	13

). Among the 37 patients who received radical surgery after chemotherapy, 16 (50%) had a failed initial laparotomy. At a median follow-up from the start of the treatment of 48 months (range 30–60 months), 25 of the 37 resected patients (68%) were alive and 24 (65%) were disease free, the 4-year survival rate for the whole group was 31%. The median survival was 17 months for the whole group and 12 months for inoperable patients, while it was not reached in resected patients (Figure 2

**Figure 2:**
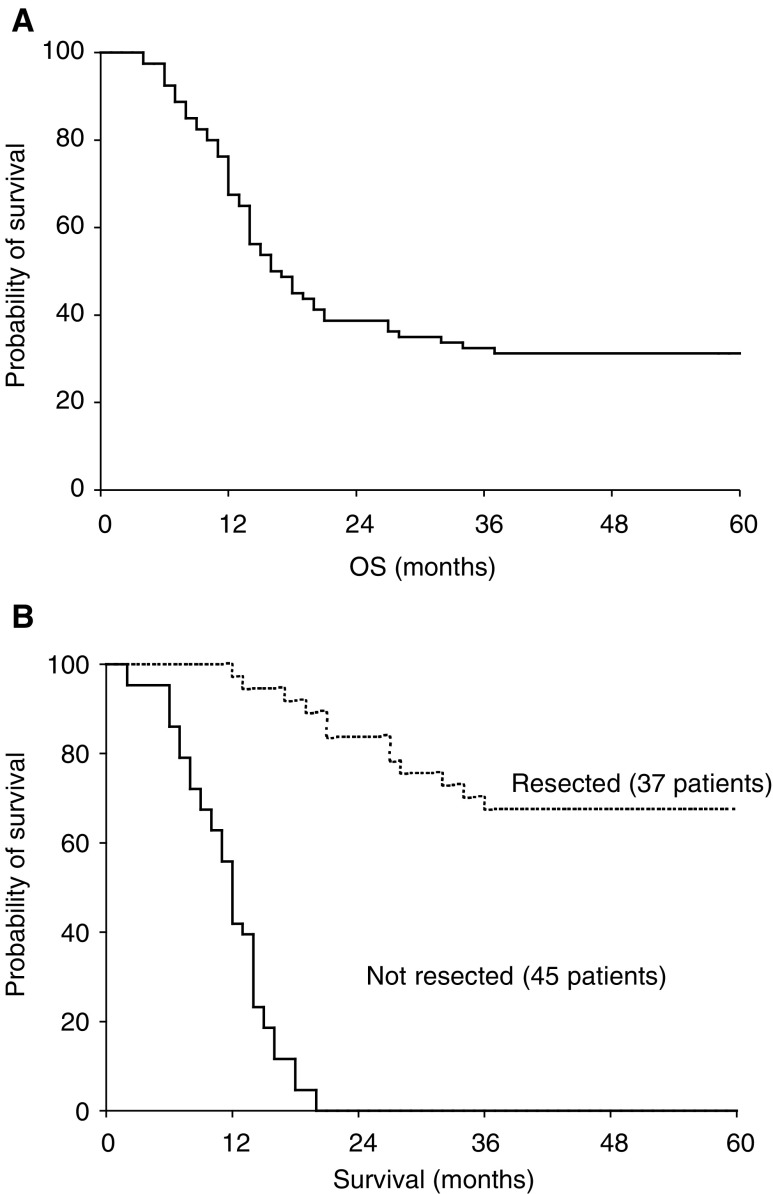
(**A**) Kaplan–Meier overall survival (OS) curve for the whole group of 82 patients. (**B**) Kaplan–Meier survival curves for patients who underwent curative resection of primary gastric tumour after chemotherapy (resected, - - - - -), and for not resected patients (not resected, ———).

). There were no deaths associated with chemotherapy or major surgical complications in this study and the toxicity profile was globally acceptable. We observed mainly haematological toxicities of grade 2: leucopenia and thrombocytopenia in five and six patients, respectively, while grade 3–4 haematological toxicities were rare events (three cases of grade 3–4 leucopenia and two cases of grade 3–4 thrombocytopenia). Nonhaematological toxicities were uncommon and moderate (Table 3

**Table 3:** Treatment toxicity (NCICTC): worst toxicity per patient

	**Grade I**	**Grade II**	**Grade III**	**Grade IV**
Leucopenia	6	5	2	1
Thrombocytopenia	5	6	1	1
Anaemia	5	3	2	—
Mucositis	4	3	—	—
Diarrhoea	—	2	—	—
Nausea/vomiting	6	10	1	—
Neurotoxicity	1	2	—	—

NCICTC=National Cancer Institute common toxicity criteria.

). Dose delays and dose reductions were applied in 25 (30%) and seven (8.5%) patients, respectively. No unplanned admissions were required at any stage during the course of chemotherapy. All patients considered suitable for surgery after chemotherapy underwent laparotomy within 6–8 weeks (median 48 days) from the end of chemotherapy, accordingly to what was originally planned in the protocol. The median hospital stay for surgery was 10 days (range 8–20 days), accordingly to what was expected. We did not observe relevant 30-day medical or surgical complications and no patients required return to operating Theatre.

## DISCUSSION

Advanced gastric cancer patients are a heterogeneous population and at least two different clinical conditions should be considered if the best treatment approach is to be defined. Patients with metastatic tumours often present in poor general conditions and with several disease-induced symptoms. In this subset of patients, a palliative treatment is a reasonable option and a careful evaluation of the therapy-related toxicity is of primary relevance in the selection of the appropriate chemotherapy regimen. On the other hand, many patients present with a locally advanced disease not amenable of a radical resection, but without metastatic involvement of distant sites. These patients are reported to have a longer survival and a globally better prognosis (Lowy *et al*, 1999; Yano *et al*, 2002). Furthermore, they are potential candidates for a treatment with curative intent, as an objective response to chemotherapy may allow a radical resection of the tumour. In these latter cases, an active chemotherapy regimen should be preferred even when this implies a greater risk of moderate–severe toxicity.

We investigated the role of an intensive weekly chemotherapy regimen in 82 patients diagnosed with unresectable gastric cancer. The overall response rate to chemotherapy in our series was 49% (40 of the 82 patients), which compares well to that achievable with other active chemotherapy regimens available (Findlay *et al*, 1994; Louvet *et al*, 2002). However, the most interesting data arising from our study is the curative resectability rate, which we obtained in this particularly difficult setting of patients: in fact, 45% (37 of the 82 patients) of all cases treated could undergo a radical resection, a procedure that was excluded at presentation. Moreover, the 4-year survival rate of the resected group was definitely higher (68%) compared to that achieved by the whole group (31%). These data seem to confirm that complete resection of all gross disease with negative margins on pathology examination represents the only potentially curative therapy for gastric cancer, and that a preoperative, effective, chemotherapy regimen might probably improve the outcome of patients, who would have been otherwise excluded from curative resection on the basis of initial findings. The chemotherapy combination we used also showed a favourable profile of toxicity, which was mild and quite acceptable. Overall, only two cases of grade 4 haematological toxicity (one case of leucopenia and one case of thrombocytopenia) were reported and no surgical complications were observed in those who underwent surgery. Other authors explored preoperative chemotherapy in locally advanced gastric cancer patients with contrasting results, basically depending on the patients’ population investigated (resectable or not resectable at presentation) and the chemotherapy regimen employed. The available data with the ECF regimen (epi-ADR, cisplatin and 5-FU) in this setting are disappointing. In a small trial by Melcher *et al* (1996), only one of 10 patients found to have locally advanced, unresectable, disease proceeded to radical surgery after ECF chemotherapy, and in a more recent study other authors substantially confirmed the findings previously reported by Melcher using the same regimen, as none of the four patients with initially unresectable disease was rendered resectable; moreover, none of them was alive at a median follow-up of 30 months (Geh *et al*, 2000). Even though it is possible that chemotherapy duration, 12 weeks as opposed to the 24 weeks of the Royal Marsden Hospital's experience, could have negatively affected results, these data do not seem encouraging for further studies in this group of patients. Interesting results have been initially reported with the use of the FAMTX regimen (5-FU, doxorubicin and methotrexate). In patients with high risk, but potentially curable, gastric tumours, 34 of 56 patients (61%) received a curative resection, but at the cost of substantial toxicity (mainly neutropenic fever) with one chemotherapy-related death (Kelsen *et al*, 1996). Nevertheless, data from a small randomised trial that compared the use of FAMTX as preoperative therapy before surgery *vs* surgery alone in operable gastric cancer could not demonstrate that the FAMTX regimen was suitable as neoadjuvant chemotherapy, and the authors concluded that more active regimens should be tested in further randomised studies (Songun *et al*, 1999). It should be noticed that in preoperatively unresectable patients, a combination chemotherapy containing methotrexate and 5-FU was reported by Plukker *et al* (1991) to allow 40% of curative resections. In a further trial, 34 patients with unresectable gastric cancer were treated with either of the two neoadjuvant chemotherapies: FEMTXP (5-FU, epirubicin, methotrexate, cisplatin) or THP-FLPM (pirarubicin, 5-FU, leucovorin, cisplatin, mitomycin C). Of 33 evaluable patients, only eight (24%) curative resections could be performed; interestingly, at multivariate analysis salvage surgery was found to be the only independent prognostic factor in this series (Yano *et al*, 2002). The EAP regimen (etoposide, doxorubicin and cisplatin) obtained more than 70% of curative resections in patients with high-risk, locally advanced gastric cancer. Nevertheless, toxicity was considerable and all cases were theoretically resectable at presentation (Schuhmacher *et al*, 2001). Results with the same regimen in not resectable cases are clearly less appealing and approached a 44% of resectability rate (Wilke *et al*, 1990). A major matter of debate in this area is also accurately defining preoperative staging procedures. Although a correct diagnosis of the extent of disease is of paramount importance in locally advanced gastric cancer, standard techniques are still to be defined. Direct vision by surgical exploration might represent the best initial assessment available, but would significantly contribute to increase treatment morbidity and might cause the patients to undergo an unnecessary laparotomy. Laparoscopy, EUS or a combination of these two techniques could complement traditional staging methods and provide clinical data not otherwise obtainable (Ajani *et al*, 1999). Demonstration exists that these procedures can improve staging of the primary tumour and detect unsuspected metastatic disease with interesting sensitivity and specificity, thus allowing a better patient selection and confrontation between different series. Particularly promising seemed data with regard to the use of EUS in preoperative staging of stomach tumours (Lightdale *et al*, 1989; Botet *et al*, 1991). Endoscopy and endoscopic ultrasonography showed an accuracy of 75–85% in predicting tumour stage before surgery, but its use in the evaluation of response to treatment in patients, who received preoperative chemotherapy, was disappointing and unreliable probably due to chemotherapy-induced changes in the physical pattern of the tumour (Kelsen *et al*, 1996; Ajani *et al*, 1999). Nevertheless, before a more widely diffusion of these methods occur, traditional procedures are to be applied in order to define surgical resectability. CT scan proved to possess an accuracy of 80–90% in defining local diffusion in gastric cancers. Clear CT scan signs of tumour extension to pancreas, aorta, omentum, oesophagus and liver, and the presence of bulky tumour (>7 cm) should discourage surgery, as initial treatment in these patients, as the probability to obtain a radical resection is very low (Sussman *et al*, 1988; Rougier *et al*, 1994). In our trial more than a half of the patients were considered not resectable by direct surgical exploration: in fact, 52 (63%) patients underwent laparotomy, as a part of a failed attempt at radical primary surgery, before study entry. In the remaining 30 (27%) patients, the diagnosis of locally advanced disease was confirmed by CT scan of the abdomen (23 patients) and EUS (seven patients). These data demonstrate that in our series preoperative tumour upstaging is unlikely to have occurred, thus making our findings fully interpretable.

Chemotherapy with weekly CDDP, epi-ADR, 5-FU, 6S-leucovorin, glutathione and bone marrow support (filgastrim) seemed to be highly effective in patients with locally advanced, unresectable, gastric cancer, as it allowed a potentially curative resection in 45% of the cases we observed. Moreover, even though the study design could not allow any definitive conclusion, the median survival and the 4-year survival time (68%) of the resected group were suggestive of a survival advantage in this subset of patients determined by the use of preoperative chemotherapy. These findings are even more interesting when we consider the favourable profile of toxicity and the short period of treatment requested (8 weeks as opposed to 12–24 weeks of other chemotherapy regimens). Taken together, the data obtained from the present study suggest that this intensive weekly regimen could represent a therapeutic option for patients with locally advanced, unresectable, gastric cancer, with the aim to allow a curative resection, and hopefully a prognostic improvement, in about half of all cases. According to these findings, the PELF weekly regimen has now become our standard ‘off-trial’ treatment for selected cases, showing clinical characteristics similar to those outlined in our protocol. However, in the daily clinical practice, this chemotherapy regimen should not always be considered a possible choice, especially for patients in poor general conditions, thus making this treatment unsuitable for approximately 15–20% of all cases. Further, well-designed randomised studies are clearly warranted in order to confirm our findings. We believe that a randomised clinical trial investigating the PELF weekly regimen against a docetaxel-containing regimen, as proposed by Ajani *et al* (2003),would be of particular interest in order to improve the standard of care in this setting.
